# A Novel In Silico–Ex Vivo Model for Correlating Coating Transfer to Tissue with Local Drug-Coated Balloon-Vessel Contact Pressures

**DOI:** 10.1007/s10439-024-03634-6

**Published:** 2024-12-12

**Authors:** Efstathios Stratakos, Linnea Tscheuschner, Lorenzo Vincenzi, Edoardo Pedrinazzi, Fragiska Sigala, Luca D’Andrea, Dario Gastaldi, Francesca Berti, Abraham Rami Tzafriri, Giancarlo Pennati

**Affiliations:** 1 Laboratory of Biological Structure Mechanics, Department of Chemistry, Materials and Chemical Engineering “Giulio Natta”, Politecnico di Milano, 20133 Milan, Italy; 2 Department of Vascular Surgery, National and Kapodistrian University of Athens, 15772 Athens, Greece; 3 Mechanics of Biological and Bioinspired Materials Laboratory, Department of Aerospace and Mechanical Engineering, University of Liège, Quartier Polytech 1, Allée de la Découverte, 4000 Liège, Belgium; 4 Department of Research and Innovation, CBSET Inc., Lexington, MA 02421 USA

**Keywords:** Drug-coated balloon, Benchtop experiment, In silico simulations, Coating transfer, Contact pressure, Drug delivery

## Abstract

Drug-coated balloons (DCBs) aim to deliver drug-loaded surface coating upon inflation at specific vascular sites, yet the role of inflation pressure remains to be defined. We implement a new approach combining ex vivo stamping experiments with in silico simulations to study acute coating transfer by commercial DCBs. This methodology comprises 3 essential pillars: (I) DCB resin inflation and slicing into cylindrical segments for subsequent stamping onto porcine-excised tissue, (II) Numerical inflation of a full DCB replica in an idealized porcine vessel to predict in vivo interfacial contact pressures (CPs) and subsequent interfacial-level numerical stamping to calculate appropriate benchtop forces that recreate these in vivo CP values, and (III) ex vivo stamping experiments and optical analysis of the stamped surfaces (DCB segment and arterial tissue), using a standard high-resolution camera to visualize coating. High-performance liquid chromatography (HPLC) was employed as a validated assay for quantifying drug in tissue samples post-stamping. HPLC analysis revealed a significant correlation with image processing, confirming the validity of the optical method as a tool to quantify DCB coating. Image and HPLC analysis revealed a statistically significant twofold rise in coating area and drug content to tissue, respectively, when the average CP roughly doubled (0.16–0.35 atm) and a non-statistically significant increase in coating area and drug content with a further rough doubling of average CP (0.35 to 0.75 atm). Imaging of DCB segments pre- and post-stamping showed transfer of partial coating thickness at low CP, contrasting with complete transfer at high CP at the same site. 3D confocal images of DCB surfaces revealed variable thickness in the transferred coating. This study introduces a comprehensive methodology for evaluating the efficacy of commercial DCB coating transfer to arterial tissue—a crucial precursor to drug elution studies—while minimizing the number of DCBs needed and improving variable control and realism.

**Supplementary Information:**

The online version contains supplementary material available at 10.1007/s10439-024-03634-6.

## Introduction

Peripheral artery disease (PAD) is a cardiovascular condition characterized by narrowed peripheral arteries due to plaque buildup, leading to decreased blood flow, while its prevalence increased from 65.8 million individuals in 1990 to 113.4 million in 2019 [1]. Drug-eluting stents (DES) and drug-coated balloons (DCBs) have demonstrated superiority over alternative PAD treatments. DES, being permanent implants, deliver antiproliferative drugs to the target lesion to prevent vessel restenosis. Meanwhile, uniformly coated DCBs offer the potential of uniformly delivering a drug-coating layer to the vessel wall with short inflation times of 0.5–3 min, allowing adaptive remodeling, multiple balloon use, and sustained drug release, while aligning with the “leave nothing behind” strategy [2]. On the downside, they present potential limitations such as particulate generation, off-target excess drug deposition, drug loss during transit, and systemic exposures [2].

Despite some concerns for the safety of paclitaxel-coated balloons, these have waned, and in fact, the use of DCBs has been increasing and expanding into various applications, including coronary vessels [3], airways [4], gastrointestinal tract [5], urinary tract [6], and the biliary system [7]. However, there remains a limited understanding of the key drivers of the achieved drug distribution patterns and their relationship to biological effects. While coatings are designed to transfer upon mechanical interaction of the DCB with the vessel wall, the roles of inflation pressure, device and vessel geometry, and tissue properties have yet to be elucidated. Understanding this relationship is crucial as both animal and benchtop studies demonstrate limited drug-coating transfer to tissue surfaces underlying the inflated DCB. Since the transferred coating serves as a distributed reservoir for sustained drug elution, it represents a key determinant of drug distribution at the target site that is amenable to optimization via device design.

Specifically, benchtop experiments of in vitro DCB inflations revealed low drug transfer to vessels [8, 9], ranging from 8 to 40 % of the initial drug load of the balloon. Correspondingly, imaging of naïve pig arteries 0.5h post in vivo treatment with commercial DCBs revealed sparse distribution of transferred drug coating onto the endoluminal surface [10], with coverages ranging from 10.2 to 17.2 %. The absence of coating transfer to the proximal and distal edges of the treatment site was correlated with reduced radial pressure at those sites.

This along with benchtop studies that have correlated coating transfer from coupon onto ex vivo tissue [11–13] suggest that development of an experimentally grounded computational model of contact pressure (CP) gradients during balloon-tissue contact may help to understand and optimize coating transfer patterns. As a first step toward achieving this goal, we recently leveraged a 3D computational model to predict interfacial CP gradients during balloon inflation in idealized vessels and assess their sensitivity to device design and procedural parameters such as balloon-to-artery ratio or inflation pressure [14]. Model simulations revealed heterogeneities in interfacial CP due to the unfolding process of DCBs and nonuniform balloon thickness that resemble coating transfer patterns in animal studies [10]. Despite these encouraging findings, a direct correlation of CP with coating transfer efficacy is yet to be elucidated, suggesting that coating transfer is not simply proportional to CP, and that heterogeneities in DCB coating must be accounted for. Therefore, in this study, we set out to experimentally evaluate the dependence of coating transfer on contact pressure for a commercial device under controlled conditions. As DCB inflation in excised and in vivo arteries does not allow for predictable control of the pressure distribution, we opted for a benchtop approach. Prior benchtop studies have examined the transfer of DCB-like coating from flat, in-house coated DCB coupons after force-controlled stamping onto excised pig arteries [11–13, 15–17]. While confirming a role for contact pressure, these approaches lack realism in replicating the 3D interfacial interaction between commercial DCB and in vivo vessels.

To overcome the limitations of existing benchtop models, the current study aimed to evaluate the feasibility of a hybrid in silico and ex vivo pipeline, previously conceptualized [18], and refine it to investigate acute drug-coating transfer by employing controlled segment stamping of a specific commercial DCB (employed as a proof of concept) onto porcine-excised tissue and subsequent optical analysis of the post-stamping surfaces. While our earlier work focused on conceptualizing this approach, the current study advances it by implementing detailed experimental protocols, integrating prior and new simulations with benchtop experiments, and introducing novel imaging and analysis techniques, all to investigate the impact of controlled experimental conditions that mimic in vivo mechanical interactions on acute coating transfer.

## Materials and Methods

### Conceptual Overview

The present methodology is based on three essential pillars, in which ex vivo experiments are combined with in silico modeling of the entire device and the local DCB-tissue interface.

**Fig. 1:**
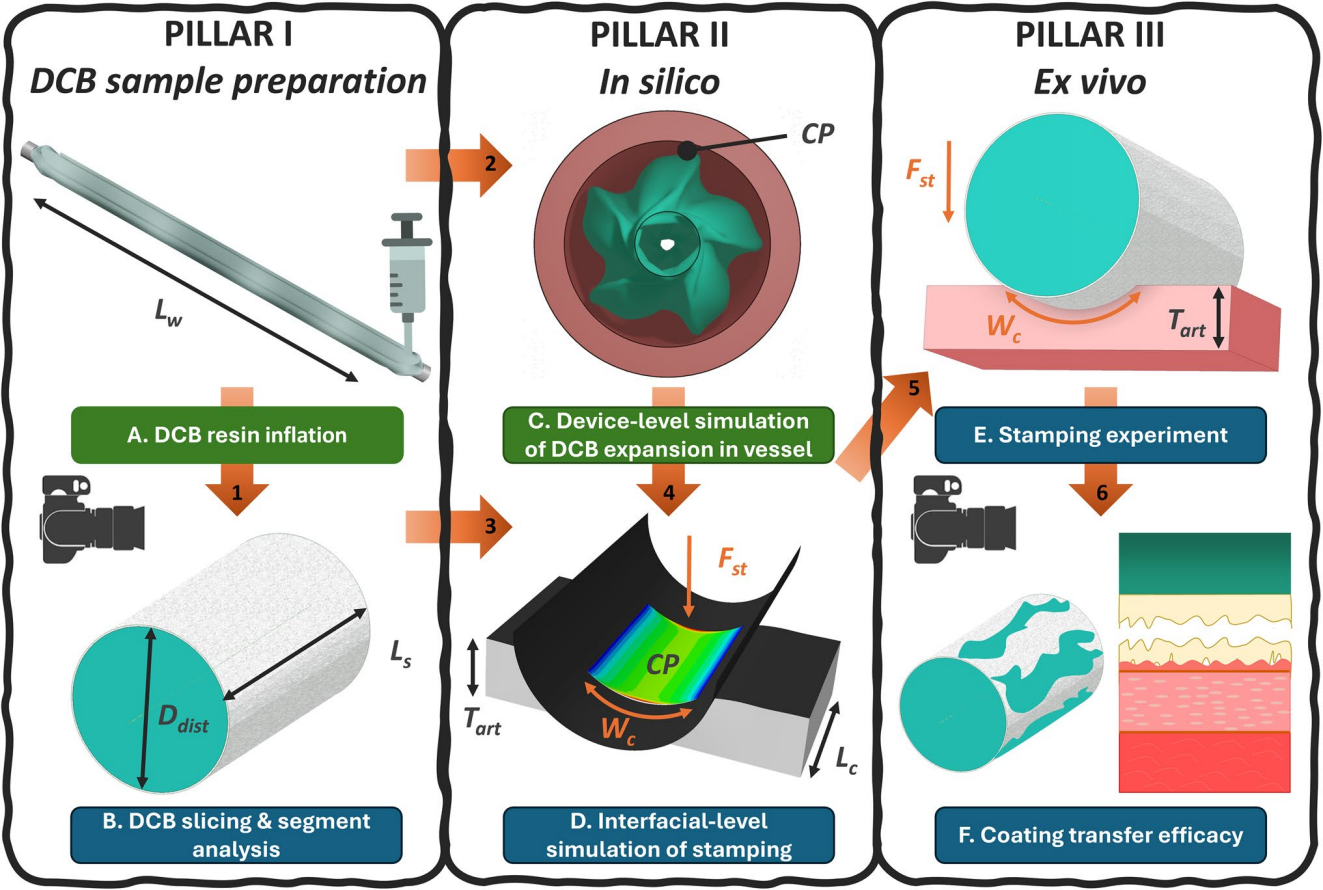
Pipeline of the in silico–ex vivo approach where green labels signify device-level and blue labels signify interfacial-level, while arrows signify the links between the different steps: **A** resin inflation of the DCB device (L_w_: DCB working length), **B** slicing of DCB into cylindrical segments (D_dist_: Distended DCB segment diameter, L_s_: DCB segment length), **C** digital replication of the full DCB device expansion in an idealized numerical vessel to deduce the CP values at NP (CP: DCB-tissue interfacial CP), **D** numerical replication of the stamping experiment to deduce the compression forces that result in the in vivo CP (F_st_: Stamping force that creates CP, W_c_: Contact width, T_art_: Arterial thickness, L_C_: Artery length), **E** the stamping experiment aiming replication of the in vivo balloon expansion by performing uniaxial compression between a DCB segment and pig-excised tissue, and **F** investigation of acute coating transfer by optical analysis on the DCB segment and the artery, as a function of experimental inputs

PILLAR I involves the production of a large number of DCB samples for subsequent stamping onto arterial patches: it entails utilizing commercial DCBs with a designated working length L_w_ as illustrated in Fig. 1A, to be inflated with a polymeric resin up to their distended diameter D_dist_ and (Link 1) section them upon solidification into cylindrical DCB segments N_s_ of length L_s_ (Fig. 1B). A minimum length *L*_s_ of 10 mm is necessary to prevent damage to the drug-coating area to be tested, during lateral slicing or mounting, rendering the two segment ends unsuitable for stamping. During the stamping experiments, these cylindrical segments can be rotated around their axis to exploit different drug-coating portions N_p_ for the interaction with the arterial patches. Hence, N_s_ × N_p_ stamping tests can be performed using a single DCB. To properly design the stamping experiments, knowledge of drug-coating distribution on lateral surfaces of DCB segments is mandatory. As commercial DCBs are coated before or after folding, coating may be distributed along the entire surface or limited to exposed stripes. Further heterogeneities can arise from manufacturing artifacts or sample preparation artifacts.

PILLAR II involves two in silico analyses at different scales. First, through a finite element replica of the full DCB device, geometrical information from the DCB inflation is acquired, and (Link 2) through the simulation of its expansion into arterial vessels, the induced interfacial CP fields during in vivo DCB inflation are estimated. By modulating parameters such as DCB-vessel oversizing, vessel stiffness, and inflation pressure (Fig. 1C), different scenarios can be investigated for a specific DCB design. Then, a numerical replication of the stamping experiment is performed (Fig. 1D) to derive two key quantities: (i) the benchtop compression forces F_st_ necessary for generating average in vivo predicted CPs during the stamping experiments and (ii) the DCB segment-vessel contact width W_c_, which affects the N_p_, i.e., the maximum number of stamping experiments for each DCB segment. This step requires information from the DCB sliced segments (Link 3) and device-level DCB expansions in vessel (Link 4) to setup the numerical simulation of benchtop replication.

PILLAR III includes the mesoscale stamping tests (Fig. 1E) and the subsequent optical analysis of the experimental outcomes. A mechanical testing machine is integrated with an ad-hoc system for gripping the DCB segments and the arterial patches and compressing each other with F_st_ forces, as deduced by the stamping simulation (Link 5), performing controlled stamping experiments at the desired CPs. Consistently with the adopted *L*_s_, arterial patches with a length of 5 mm L_c_ are used to interact only with the central portion of DCB segments. Potentially, N_p_ stamping tests can be performed for each DCB segment, by suitably rotating the sample around its axis. Images are collected with the high-resolution camera to evaluate the drug-coating distribution on the DCB segment (both before and after stamping), as well as the drug coating transferred onto the tissue (post-stamping arterial surface) (Link 6). Finally, classical image segmentation is used to quantify the coated areas and then assess the acute transfer efficacy of the devices as a function of the investigated parameters (Fig. 1F).

PILLARS I–III leverage standard accessible methodologies that can be coupled to additional assays for enhanced insights. Here, we demonstrate the enhancements obtained from two additional measurements: (i) high-performance liquid chromatography (HPLC) analysis of stamped arteries to quantify net drug transfer and (ii) confocal laser microscopy to deduce the microstructural aspects of the drug coating onto the DCB full device prior to processing and onto the artery post-interaction with the DCB segments. These methodologies are excluded from the essential pillars of the suggested approach due to the need for advanced experimental equipment. However, they can optionally provide more sophisticated analyses to complement the DCB assessment.

### Devices

For our analyses, two commercial DCBs (under the name Pearlflow, L2MTech GmbH) were provided. Specifically, they had 6 mm nominal diameter at 7 atm of nominal pressure NP, an *L*_w_ of 60 mm, and five folds. As indicated in the package label, the device incorporates 3538 μg of paclitaxel at a nominal dosage of 3.0 μg/mm^2^ and a Butyryl-tri-hexyl citrate (BTHC) matrix.

DCBs can be coated either in a folded or distended state. Coating in a distended state ensures uniform drug distribution but may lead to drug loss during folding. Conversely, coating in a folded state minimizes the risk of drug loss during delivery, though achieving uniform coverage can be more challenging [19]. Most manufacturers do not disclose detailed information about their coating processes due to proprietary technology. However, it has been previously demonstrated that for Elutax 3, Elutax SV Fistula, INPact Admiral, Lutonix 035, and Stellarex, the drug coating is either folded inward or protected by an additional top or hydrophilic layer, signifying that the balloon is coated at distended state [20]. In contrast, for Luminor 35 and Ranger, the drug appears to be applied primarily to the exterior of the folded balloon signifying that they are coated at a folded state [20].

During method development, five linear coating patterns were observed on the DCBs employed in the present study, each covering an arc of W_cc_, corresponding to its number of folds and consistent with the coating application, while the device was folded.

### PILLAR I—Methods: DCB Sample Preparation

#### DCB Resin Inflation

**Fig. 2:**
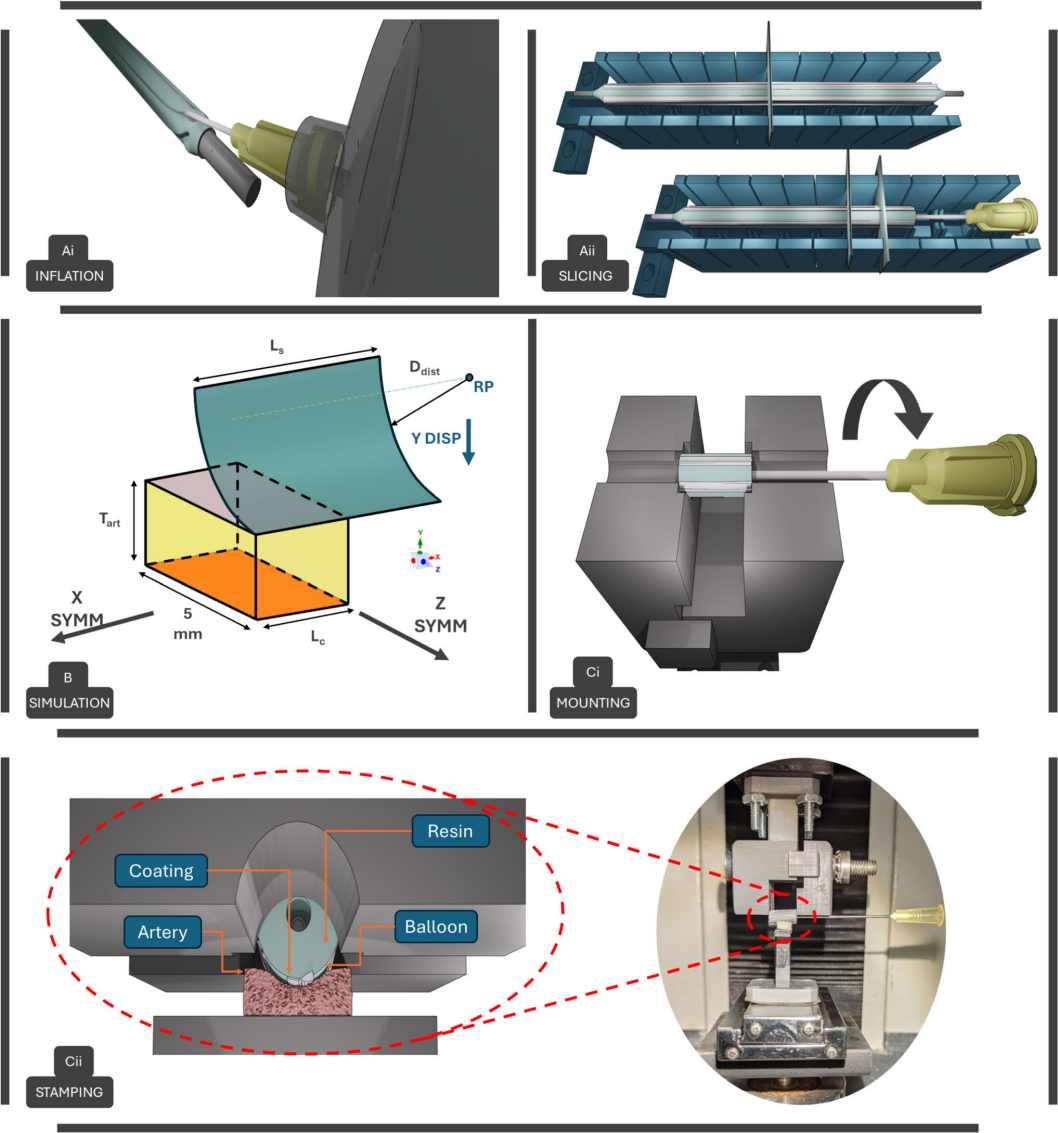
**A** DCB sample preparation and mounting: Ai Resin inflation of the DCB device using a syringe, Aii slicing of the DCB in segments exploiting an SLA printed setup. **B** Boundary conditions for the numerical simulations of DCB stamping: Symmetry conditions were applied at the two front (yellow) surfaces of the arteries and the bottom (orange) surface was encastered. A reference point controlled the vertical displacement of the rigid DCB segment replica until the required pressure was achieved. **C** Experimental protocol: Ci. Mounting of DCB segments onto the grips and imaging (rotation of the DCB allowed for testing of multiple coated areas) and Cii Left: Compressing the DCB segment onto the pig-excised tissue, and Right: Experimental apparatus

As direct slicing of DCB pieces for stamping is not feasible without damaging the drug coating, a strategy involving resin injection was developed to create rigid DCB segments. Fast-curing polyurethane resin (RESIROCK, Hobbyland), which polymerizes upon interaction of its two components, was used. However, the high viscosity of the resin rendered the inflation port as ineffective, as it encountered substantial resistance over the extended distance from the port to the balloon. Consequently, an alternative inflation technique was devised and applied to two of the DCBs. Under a biological hood, the DCBs were unsheathed, and a 20 G needle was used to pinch its proximal part of the balloon membrane, ensuring needle insertion up to the catheter marker. Super glue was employed to secure the connection, with parafilm wrapped around the needle insertion point to prevent resin leakage. A stopcock was then connected to the needle, and a stainless-steel wire was passed through the guidewire port to maintain balloon alignment, with the distal end secured in a gripping system. The two polyurethane components, mixed with color powder to increase background for coating visibility, were then applied to the balloon using a 5-mL syringe, with a prior vacuum applied to remove residual air. Subsequently, the syringe was filled with the resin mixture and attached to the needle, and DCB inflation was performed until the balloon reached full distension with a circular shape (Fig. 2Ai). The DCB distended diameter D_dist_ was then measured proximally, distally, and in the middle section of the DCB; moreover, the external surface of the DCB segments was imaged using a high-resolution camera (Canon EOS 6D) with a macro-objective (Canon MP-E 65mm f/2.8 1–5X) to obtain information about the drug-coating distribution.

#### DCB Specimen Slicing and Imaging

A stereolithography (SLA) -printed setup was devised to aid the careful and precise sectioning of DCB segments (Fig. 2Aii). This was manufactured by employing a photosensitive polymer resin (Gray resin, Formlabs) with a resolution of 50 µm in the xy-plane. The setup featured multiple supports and slots, providing resistance, and allowing the passage of a blade. The tested specimens aimed to have an overall length *L*_s_ of 10 mm, acquiring 6 DCB segments from each device. Therefore, blade slots were set at 10 mm of distance from each other. Slots and supports measured 0.3 mm and 1.5 mm in length, respectively. The arc of the hinges corresponded to the diameter of the DCB D_dist_ at its resin-inflated configuration. Fillet application on the supports minimized contact. To permit resin solidification, 25 min following inflation, the DCB was positioned on the slicing setup (Fig. 2Aii). One blade was inserted halfway through the DCB diameter to maintain balloon constraint during cutting, while another blade was utilized for the slicing process. Additionally, a needle was inserted midway through the DCB diameter to control the specimen, with wooden supports placed alongside the cutting setup to absorb impact and minimize coating loss.

### PILLAR II—Methods: Numerical Simulations

#### Device-Level DCB Expansion in a Vessel

To investigate the balloon’s interaction with the arterial endolumen during DCB expansion, we implemented a previously developed finite element model DCB inflation [18] using Abaqus/Explicit (Dassault Systemes Simulia Corp, Johnston RI), to simulate the folding and unfolding of Pearlflow in a simplified arterial vessel representative of healthy arteries. After geometrical reconstruction of the DCB device and calibration of a material model based on the device’s compliance chart, the balloon was inflated inside an idealized vessel with 20% oversizing (representing a healthy animal artery with wall thicknesses consistent with pig femoral arteries (0.5 mm) [21] to obtain CP values. Vessel length was 15 mm longer than the balloon (double the length of the balloon) to ensure full containment and to avoid the influence of the boundary conditions on the developed CPs. Due to the symmetry of the numerical problem, half-symmetry conditions were adopted both for the DCB and for the vessel at the middle segment (as described in Ref [14]), considering the following BCs:

$${\text{U}}_{{3}} ,{\text{ UR}}_{{1}} \;{\text{and UR}}_{{2}} = \, 0,$$

where U_3_ denotes the displacement on the *z* axis and UR_1_ and UR_2_ denote the rotation around the *x* and *y* axis, respectively. The arterial nodes were encastered on the other end, far from the treatment zone and the DCB nodes were constrained as follows:

$${\text{U}}_{{1}} ,{\text{ U}}_{{2}} ,{\text{ UR}}_{{1}} ,{\text{ UR}}_{{2}} \;{\text{and UR}}_{{3}} = \, 0,$$

where U_1_ and U_2_ denote the displacement on the *x* and *y* axis and UR1, UR2, and UR_3_ denote the rotation around the *x*, *y,* and *z* axis, respectively. The folding process was modeled using five rigid jaws and ten pleating plates, with boundary conditions consistent with previous studies [22]. The unfolding was captured using a dynamic explicit solver, maintaining a kinetic-to-internal energy ratio below 5%, and setting the inflation time to 0.02 s. Contact modeling employed "hard" contact for pressure-overclosure and a friction coefficient of 0.2 for tangential behavior. Pressure transitions were modeled with a "smooth step" function, ranging from 0 to 1 atm during folding and increasing to 9 atm during expansion, as DCBs may be inflated at pressures above nominal. The balloon was simulated as a bilinear elastoplastic material (Young’s modulus = 1150 MPa, yield stress = 30 MPa, plastic modulus = 158 MPa), while the artery was modeled using a 5-parameter Mooney-Rivlin hyperelastic material [23] and plotted the CP distribution onto the vessel endolumen. The model assumed near incompressibility, using a Poisson's ratio of 0.475, and the vessel geometry was meshed with 206,870 elements, with a focus on higher element density in areas of balloon–vessel interaction.

#### Stamping of Resin-Hardened DCB Segments

Prior to in vitro tests, numerical simulations were conducted to reproduce the in vivo DCB-to-tissue compression and correlate the benchtop applied force F_st_ with the resultant CP exerted on the arterial tissue and contact width W_c_ (Fig. 1D). Simulations were necessary given the complexity of the cylinder-on-plane contact and boundary effects, which preclude the analytical approach. Figure 2B demonstrates the boundary conditions employed in the simulation. Given the symmetry of the problems, quarter symmetry conditions were adopted. Thus, the dimensions of the following models referred to a quarter of those of the actual samples utilized in the experimental phase. A quarter-cylinder surface replicated the DCB model, which mimics the dimensions of the DCB segment in its distended configuration. Mechanical properties were modeled through a discrete rigid body, due to its high stiffness compared to the arterial tissue. The balloon was meshed with 4-node 3D bilinear rigid quadrilateral elements (R3D4) and consisted of 2200 elements. The artery model’s geometry was derived from the average dimensions measured from various artery samples of the pig aortic arch. The physical properties of the artery have been approximated as those of an elastic isotropic material with Poisson's ratio *ν* = 0.17, mass density *d* = 1.07 g/cm^3^, and Young's Modulus *E* = 125 kPa [24, 25]. The artery geometry was meshed with 25000 8-node linear brick elements with reduced integration (C3D8R). The dimensions of the instances and their boundary conditions are seen in Fig. 2B. An implicit dynamic solver with a time period of 0.001 s was employed for the compression step. During that step, displacement-controlled vertical movement was applied to the quarter cylinder through a reference point (RP). The reaction forces at the RP were analyzed during the frames of the compression step and were correlated with the average CP at contact points. To examine the impact of arterial thickness and stiffness variability on F_st_, CP, and W_c_ values, we conducted four additional simulations, adjusting each parameter by ± 20% with respect to the control values. The outputs of the simulation were imported into MATLAB to plot the CP distribution onto the arterial tissue at different compression levels.

### PILLAR III—Methods: Benchtop Stamping and Analysis

#### Testing Setups

A custom apparatus was designed, and 3D printed to securely hold specimens during testing (Fig. 2Ci). The DCB segments were gripped laterally and placed on a hinge to maintain consistent sample positioning. The resin-embedded needle was exploited for sample manipulation and rotation to align the coating stripe parallel to the arterial endothelium during compression. Compression of the apparatus, and consequently lateral gripping of the DCB specimen, was achieved using a screw and a nut. Subsequently, the DCB segment was imaged with the camera (Fig. 2Ci) and mounted onto a uniaxial testing machine. The artery model was positioned on a bed, connected to the bottom part of the testing machine (Fig. 2Cii). The equipment enabled lateral needle insertion, thereby allowing the DCB segment to be rotated between each compression to test different parts of the segment.

#### Artery Preparation

For the arterial samples, the aortic arch and descending aorta of two swine, kindly provided by a local abattoir, were cleaned from surrounding fat and fascia. Arterial sections with approximately the same thickness of 2 (± 0.4) mm, consistent with ex vivo measurements [26], were cut into specimens with a width of 5 mm and a length of 10 mm. Prior to stamping, all arterial samples were allowed to equilibrate for at least 1 h, up to 4 h in 0.9 % NaCl solution at 37 °C, and stored there until testing.

#### Experimental Protocol

Stamping experiments (Fig. 2Cii) were performed in two compression groups using an MTS Synergie 100 tensile testing machine (100 N load cell) to simulate two distinct scenarios divided into two groups. The first group (Group 1) replicated balloon expansion without prior coating straining, by compressing the central portion L_s_/2 of the pristine DCB segment onto a tissue for a prolonged time (60 s) (Table 1), imitating solely in vivo DCB expansion [27]. The second group (Group 2) included a coating preconditioning via compression of the full-length L_s_ of the DCB segment onto the artery for a short time (2 s) at various CPs before extended compression onto another artery to mimic DCB expansion (Table 1). Preconditioning of Group 2 was designed to simulate brief interactions that DCBs may encounter prior to inflation in the target area (gloves touching the DCB or interaction of the DCB with tissue/guiding catheter during tracking).

**Table 1:** Experimental variables: Group 1 involved a single interaction of each DCB coating with tissue for 60 s at Low, Intermediate, and High average CP, and Group 2 involved an initial 2 s stamp (preconditioning tap) of the coating onto an artery at Low, Intermediate, or High average CP and subsequent stamping on another artery for 60 s at Low, Intermediate or High average stamping CP

Group 1: Pristine DCB samples stamping					
Stamping		CP	Low	Intermediate	High
		Time (s)	60	60	60

Group 2: Preconditioned DCB samples stamping					
Preconditioning		CP	Low	Intermediate	High
		Time (s)	2	2	2
Stamping		CP	Low	Intermediate	High
		Time (s)	60	60	60

All experimental variables were tested with 4 repetitions. Each DCB segment was aimed to be used for multiple stamping tests N_p_, maximizing the exploitation of pristine drug-coated areas. The number of tests per balloon was defined after DCB imaging (PILLAR I**—**evaluation of the coated area) and FE simulations (PILLAR II**—**assessment of W_c_). Preconditioning and stamping experiments were performed following the scheme in Table 1, by applying displacement (at a rate of 2 mm/min) until a certain force level was achieved (indicated by numerical simulations to recreate the desired CP) for a certain time interval (Table 1). 1.5 mm of the lateral coating portions of DCB segments (total length *L*_s_ = 10 mm) are prone to damage during slicing and mounting on the test apparatus, attributed to the interaction with the slots. These portions were excluded from stamping by using arterial samples of maximum length *L*_c_ at 5 mm (Fig. 2B).

#### Camera Imaging and Segmentation

DCB segments of Group 2 were imaged prior to and after any interaction with the tissue using the high-resolution camera (Canon EOS 6D). Arterial samples were imaged following drying under environmental conditions, as performed in an in vivo study [10], under low light conditions. Segmentation of DCB and coated artery areas was performed using semiquantitative MATLAB/ImageJ codes with manual corrections as needed. To evaluate coating transfer efficacy, two methods were used:

1. Theoretical Coating Transfer (TCT): Calculated by dividing the coating area measured onto the artery by the DCB coating stripe area (W_cc_ × L_c_). This method was applied to all tests to compare image and HPLC analysis results.
2. Measured Coating Transfer (MCT): Calculated by dividing the coating area on the artery post-interaction by the coating area on the DCB pre-interaction. Due to the increased accuracy yet time-intensive nature of this method, it was exclusively applied to two representative cases from the Group 2 tests to serve as a proof of concept.

### Additional 1—HPLC Analysis

Dried arterial samples were transferred to 1.5-mL tubes, and 1 mL of methanol and 10 µL of 10 ng/mL carbamazepine as an internal standard was added to each sample. To extract the transferred paclitaxel, arterial samples were incubated for 1 h in the solvent on a rocking shaker at room temperature. Extracted samples were stored at − 80 °C until further analysis.

After extraction, the samples were analyzed with HPLC system. Liquid chromatography system consisted of an ExionLC system (AB SCIEX, Canada), a Kinetex C18 (100 X 2.1 mm, 2.6 μm) column (Phenomenex, Germany), and a binary pump. Mobile phases consisted of 0.1% formic acid in water (Millipore) (solvent A) and 0.1% formic acid in acetonitrile (solvent B). Chromatography was performed using a gradient program starting with 80% solvent B and decreasing to 0% solvent B over a time span of 10 min and a flow rate of 0.2 mL/min. The mass spectrometry system consisted of a SCIEX QTRAP 6500+ mass spectrometer (AB SCIEX, Canada) with electron spray ionization (Turbo V ion source) in positive ionization mode, working in multiply reaction mode (MRM). Carbamazepine was used as an internal standard to minimize matrix effect and signal drift during analysis.

### Additional 2—DCB Coating Imaging with Confocal Laser Microscope

One of the two DCB devices available was prepared for device assessment by removing its sheath and positioning it under a microscope with one balloon fold oriented parallel to the lens. Utilizing confocal laser imaging technology (Olympus LEXT OLS4100 3D Measuring Microscope), quantitative surface reconstruction was conducted to determine the thickness of the drug coating within a localized region, and high-magnification (×100) imaging to assess the coating microstructure before unfolding. Post-stamping, a high-magnification confocal image of the transferred coating onto the artery was acquired to assess potential variability in the thickness and changes in microstructure.

### Statistical Analysis

To assess the statistical significance of differences in coating area and drug quantity between groups subjected to varying contact pressures, we conducted pairwise two-sample *t* tests. For each unique contact pressure, the corresponding coating area and drug quantity data were isolated. We then performed *t* tests between all possible pairs of these contact pressures, comparing the mean coating area and mean drug quantity between groups. The *t* tests were conducted under the assumption of equal variances and were used to evaluate the null hypothesis that the means of the two groups were equal. The resulting *p* values were recorded to determine whether the observed differences were statistically significant, with a significance level set at *α* = 0.05. These *p* values were subsequently visualized using bar plots to facilitate interpretation of which pairwise comparisons showed significant differences.

## Results

### PILLAR I—Results: DCB Sample Preparation

**Fig. 3:**
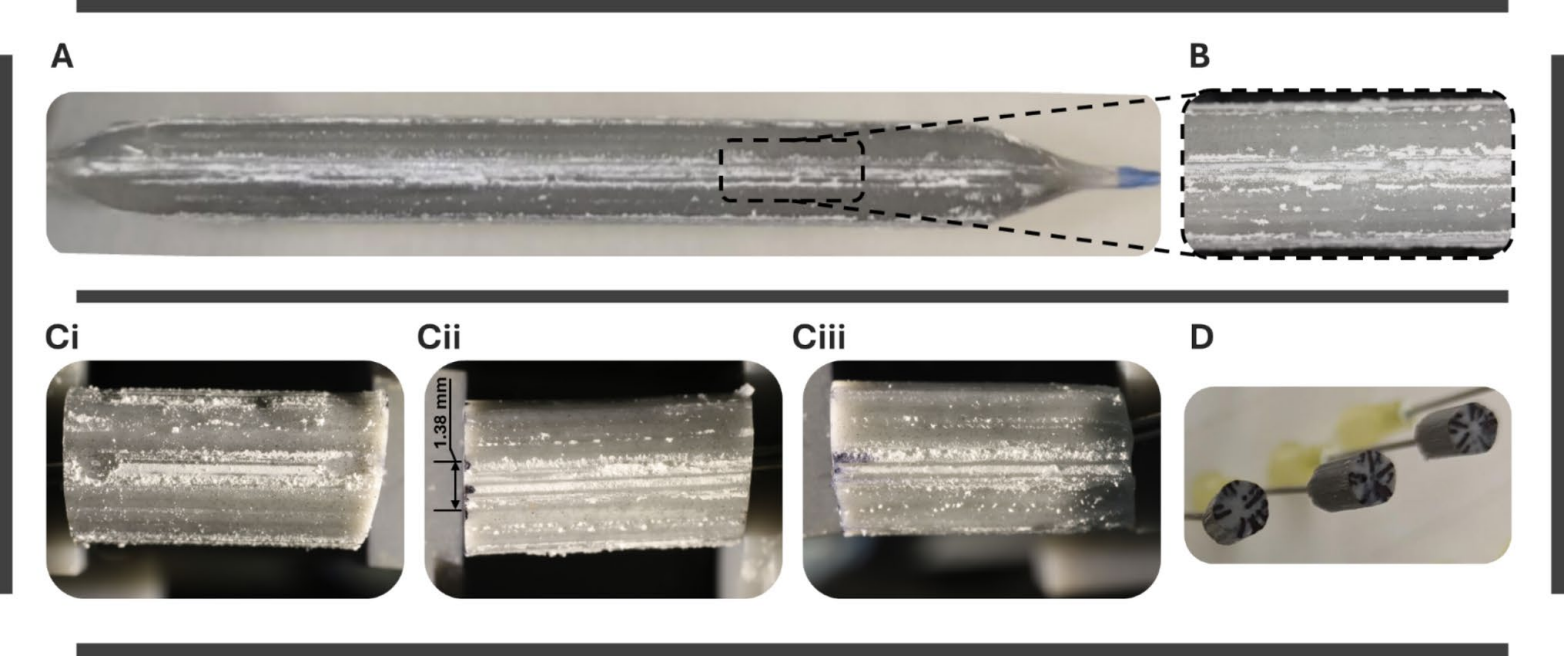
Resin-inflated DCB pre- and post-slicing: **A** resin-inflated DCB demonstrates clustered coating along stripes, **B** high-resolution camera image at a local area at the center of the DCB post-inflation, **C** various DCB segments post-slicing and mounting, and **D** profile of the DCB segments shows a roughly circular shape

Resin inflation of the DCB device demonstrated heterogeneous patterns of coating, with significant coating clustering along stripes that were themselves heterogeneous (Fig. 3A). High-magnification camera imaging of the inflated DCB revealed distinct coating lines per stripe (Fig. 3B). Post-sectioning DCB segments retained coating integrity, except for partial damage near slicing and gripping locations, which motivated performing stamping at the stripe's center (Fig. 3Ci–iii). Additionally, Fig. 3Ciii shows a lower diameter at one extremity of the DCB segment which was closer to the inflation point. The coating stripe width *W*_cc_ was measured 1.38 (± 0.21) mm (Fig. 3Ci–iii). Sliced segments showed repeatability in profile shape and dimensions at a *D*_dist_ of 5.18 (± 0.1) (Fig. 3D). Based on the *W*_cc_ and *D*_dist_, the arc of the lateral stripes *A*_ls_ was calculated at 5.13 mm.

### PILLAR II—Results: Numerical Simulations

#### DCB Inflation

**Fig. 4:**
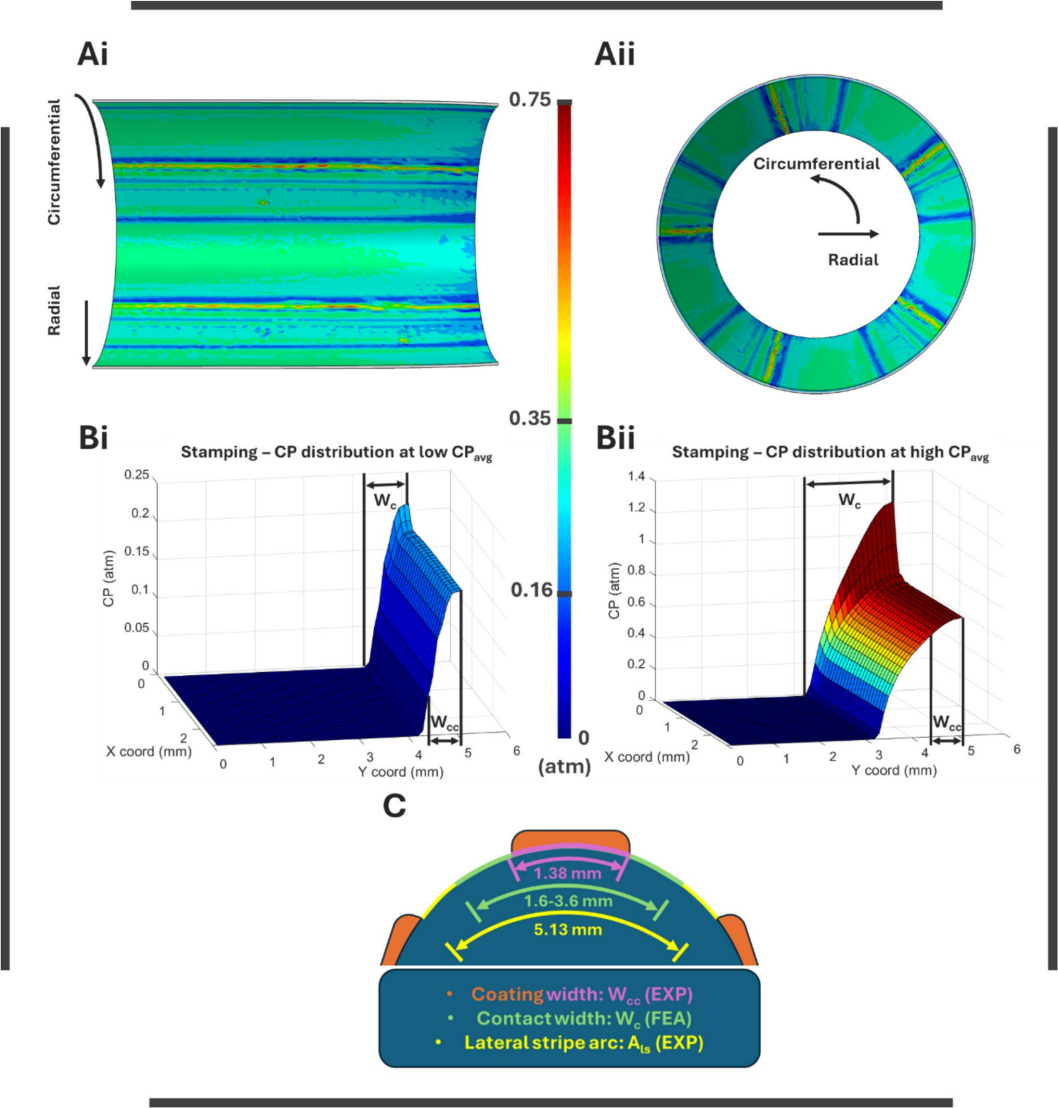
Numerical simulations and correlation with DCB segment profile: **Ai** longitudinally sliced section of the arterial endolumen showing predicted CP at 9 atm of inflation pressure and **Aii** perspective side view of the treated arterial site **Bi** predicted CP distributions for Low and **Bii** high stamping average CPs and **C** illustrative cross-section of the DCB demonstrating geometrical coating characteristics and numerically predicted interactions with the artery

The device-level numerical simulation of DCB expansion in an idealized vessel at 9 atm of inflation pressure revealed highly heterogeneous in vivo CPs, with linear patterns from balloon folds ranging from approximately 0 to 0.75 atm (Fig. 4Ai and ii). Based on these simulation results, we considered three CP average values during stamping—0.16 atm (Low), 0.35 atm (Intermediate), and 0.75 atm (High)**—**to observe their effect on acute coating transfer.

#### Stamping

Processing of interfacial-level stamping simulation and conversion of CP at nodes to 3D plots, at which the *z*-axis illustrates the amplitude of CP, for each compression load, revealed the CP distribution at the DCB-tissue interface during compression (Fig. 4B). Results revealed the required reaction forces at the RP at 0.08, 0.35, and 1 N to achieve the desired average CP values (0.16 atm**—**Low, 0.35 atm**—**Intermediate, and 0.75 atm**—**High, respectively) at the coating stripe width W_cc_ area (Fig. 4B). The total width of the DCB segment in contact was found to be 1.6, 2.6, and 3.6 mm for each respective force. Due to discontinuity at the border of the balloon with the artery, pressure concentration occurred, which amplified with increasing load (Fig. 4Bi and ii). Low CP (Fig. 4Bi) revealed steeper changes in CP when compared to high CP (Fig. 4Bii), along the W_c_ of the artery. The circumferential distance between the two lateral coating stripes A_ls_ was estimated to be 5.13 mm based on experimental measurements. Stamping simulations ensured that during all the compressions the full area of the stripe interacted with the artery and only one stripe of the DCB specimen’s coating interacted with each artery, as illustrated in Fig. 4C.

### PILLAR III—Results: Benchtop Stamping and Analysis

#### Image Analysis

**Fig. 5:**
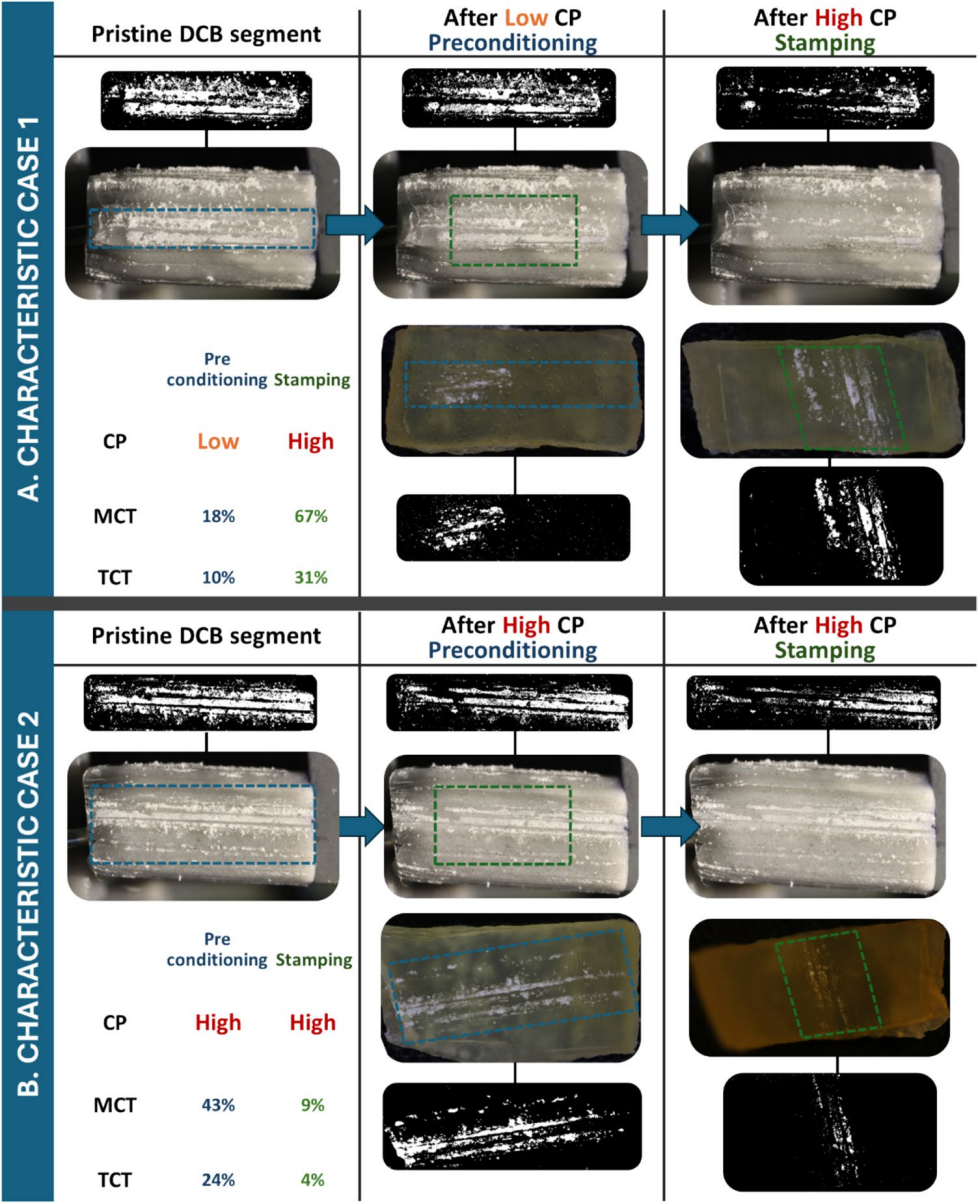
Two characteristic cases of Group 2 Preconditioning and Stamping: **A** Case 1 demonstrates that coating from the balloon is partially transferred with Low CP during preconditioning and the remaining coating is significantly transferred at high stamping CPs. Blue signifies preconditioning and green stamping. The measured coating transfer (MCT) was consistently higher than the theoretical coating transfer (TCT) (18% vs 10% for preconditioning and 67% vs 31% for stamping, respectively) as it considered the real pixel number on the DCB segment. **B** Case 2 reveals that 43% MCT and 24% TCT are transferred in a High CP preconditioning and only 9% MCT and 4% TCT of the remaining coating are transferred with subsequent High CP stamping.

In Group 2, segmentation of coating and image analysis of the DCB segment before and after interaction with the tissue allowed for correlation of the coating area on the DCB before interaction and the corresponding transfer of the coating onto the tissue (MCT). Fig. 5 demonstrates two characteristic cases of Group 2. In the first case, coating preconditioned with Low CP delivered 18% MCT and 10% TCT onto the tissue and subsequent stamping at High CP removed most of the coating from the DCB delivering 67% MCT and 32% TCT of the remaining coating area onto the tissue. Contrastingly, in the second case, pre-stamping at High CP delivered 43% MCT and 24% TCT and subsequent High CP stamping only delivered 5% MCT and 4% TCT of the remaining coating area onto the artery.

#### Image—HPLC Correlation

**Fig. 6:**
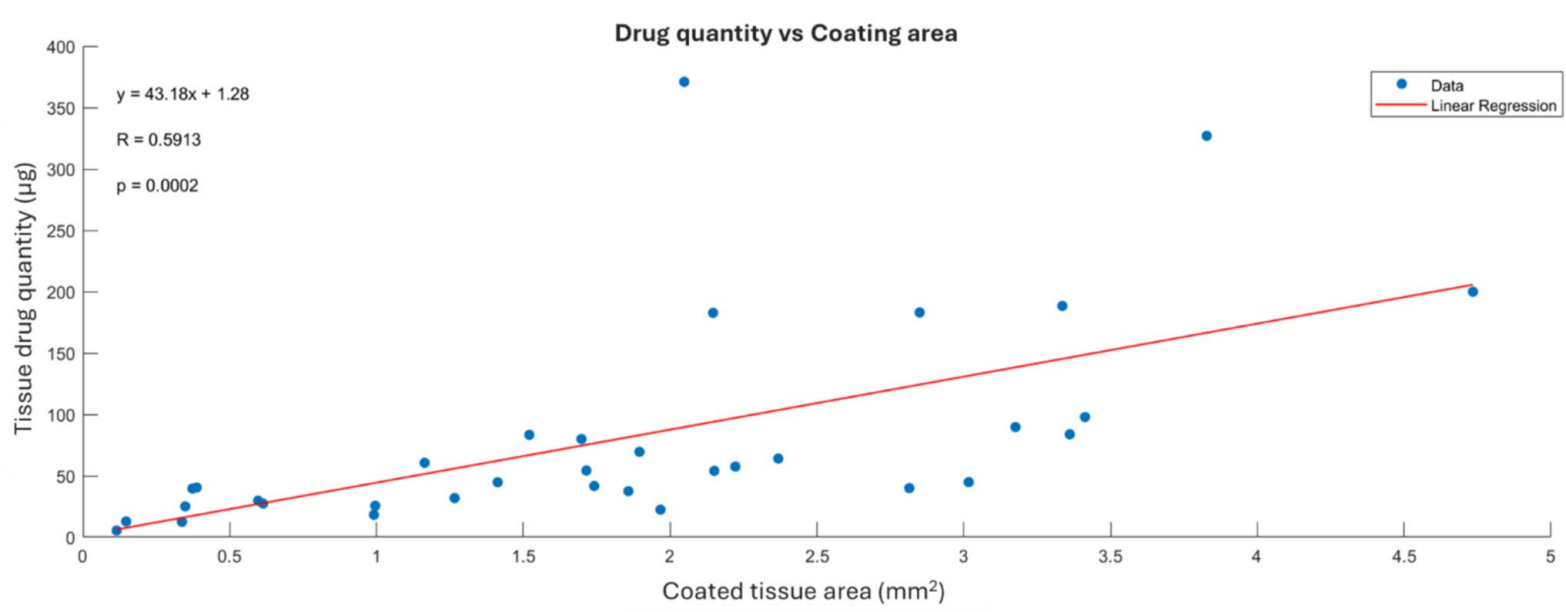
Linear regression among all data included in the study estimated a significant correlation between drug quantity and coated area.

Figure 6 demonstrates the correlation of image and HPLC analysis among all data processed both for Group 1 and Group 2. Linear regression predicted coating density onto the tissue at 43.18 μg/mm^2^ with a coefficient of determination R at 0.5913 and a *p* value at 0.0002 for the correlation coefficient.

**Fig 7:**
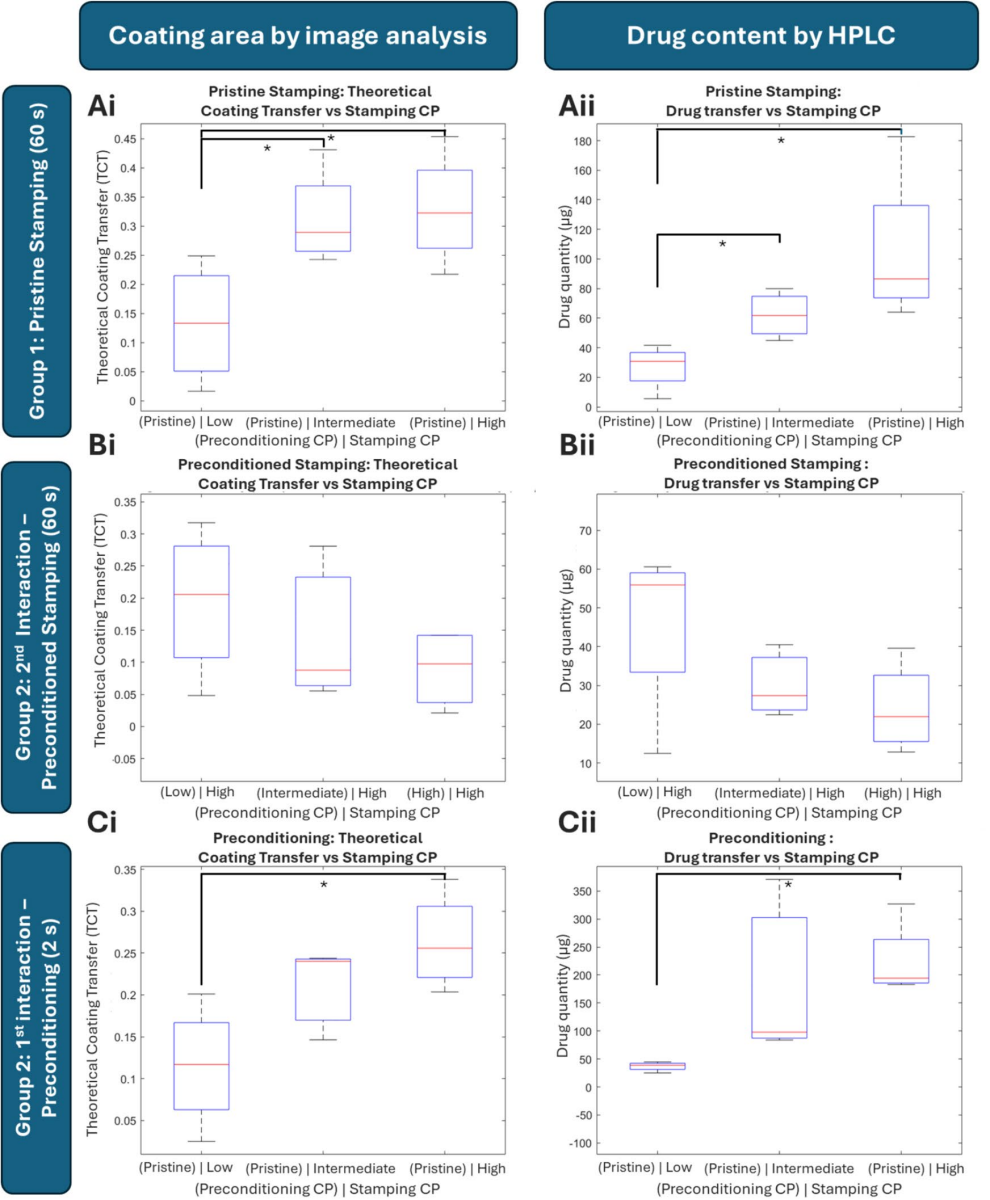
Boxplots Image & HPLC analysis in Group 1 and Group 2: Group 1**—**Stamping **Ai** linear increase in coating transfer area with average CP from Low to Intermediate CP, which is saturated with a further increase of CP, **Aii** corresponding linear increase in drug content from Low to Intermediate CP, with a further non-statistically significant 1.4-fold increase in drug content with further increase in CP. Group 2**—**Stamping **Bi** linear 2-fold decrease in coating transfer in the 2nd DCB-tissue interaction when average CP in the 1st interaction increased from Low to Intermediate, which is saturated with a further increase of CP and **Bii** 2-fold decrease in drug content in the artery involved during the 2nd DCB–tissue interaction when increasing CP from Low to Intermediate in the 1st interaction, and 1.2-fold decrease with further increase of CP in the 1st interaction. **Ci** Linear increase in coating transferred area with average contact pressure from Low to Intermediate CP, which is saturated with a further increase of CP and **Cii** Corresponding linear change in drug content from Low to Intermediate CP, and Intermediate to High

For the tests of Group 1, considering a uniformly coated stripe on the balloon at 1.38 mm of *W*_cc_ and 5 mm of *L*_c_, the maximum theoretical transfer area would be 6.9 mm^2^ (TCT). Image analysis of the camera-segmented images suggests a median range of ratios from 0.14 to 0.32 of TCT (Fig. 7Ai). Statistically significant doubling in coating transfer area was observed when average CPs increased approximately 2.1-fold, from Low to Intermediate. Further 2.1-fold increase in average CP from Intermediate to High CP resulted in a minor and statistically insignificant increase in the coating area. HPLC analysis revealed a doubling in drug content from 30 to 60 μg, when the CP roughly increased from Low to High, while further increase in CP from Intermediate to High resulted in a non-statistically significant 1.4-fold increase in drug content (Fig. 7Aii). Overall, coating segmentation showed a range of 0.1 to 3.2 mm^2^ of coating transfer, and HPLC analysis revealed a range of drug to tissue from 5 to 180 μg.

By contradistinction, stamping after preconditioning demonstrated inverse trends in coating area and drug transfer onto tissue (Fig. 7Bi and Bii). Specifically, stamping at High CP of Group 2 preconditioned DCB segments stripes onto pristine arterial tissue samples, revealed a range of median TCT ratios from 0.1 to 0.2 (Fig. 7Bi). A 2-fold decrease in coating transfer area at the 2nd artery was observed with an increase in average CP from Low to Intermediate during the interaction with the 1st artery. Further increase in CP from Intermediate to High CP on the first interaction revealed an insignificant increase in coating transfer in the 2nd interaction. HPLC data indicated a roughly 2-fold decrease in coating area onto the 2nd artery when varying CP from Low to Intermediate on the first interaction (Fig. 7Bii). Further increase from Intermediate to High CP on the first interaction showed negligible change in drug content on the second interaction. Neither image analysis nor HPLC revealed any statistically significant change in coating area or drug content for any group of pressure changes. Coating segmentation showed a median range of 0.2 to 2.2 mm^2^ of coating transfer, and HPLC analysis revealed a range of drug to tissue from 15 to 62 μg.

The preconditioning step of Group 2 DCB segments (via 2 s stamping) resulted in coating transfer trends similar to those achieved via 60 s stamping of pristine DCB. Specifically, considering a uniformly coated stripe on the balloon at 1.38 mm of W_cc_ and 10 mm of L_c_, the maximum theoretical transfer area would be 13.8 mm^2^. This suggests a median range of ratios from 0.12 to 0.25 of TCT during preconditioning (Fig. 7Ci). More specifically, a 2.2-fold increase in coating transfer area was observed with a similar increase in average CP from Low to Intermediate (Fig. 7Ci). A further increase in CP from Intermediate to High revealed an insignificant increase in coating transfer. HPLC data showed a linear 2-fold increase from Low to High CP followed by another 2-fold increase from Intermediate to High CP (Fig. 7Cii). Both image analysis and HPLC indicated a statistically significant increase only for changes from Low to High CP. Coating segmentation revealed a range of 0.1 to 4.7 mm^2^ of coating transfer, and HPLC analysis showed a range of drug to tissue from 25 to 360 μg.

#### Confocal Laser Images

**Fig. 8:**
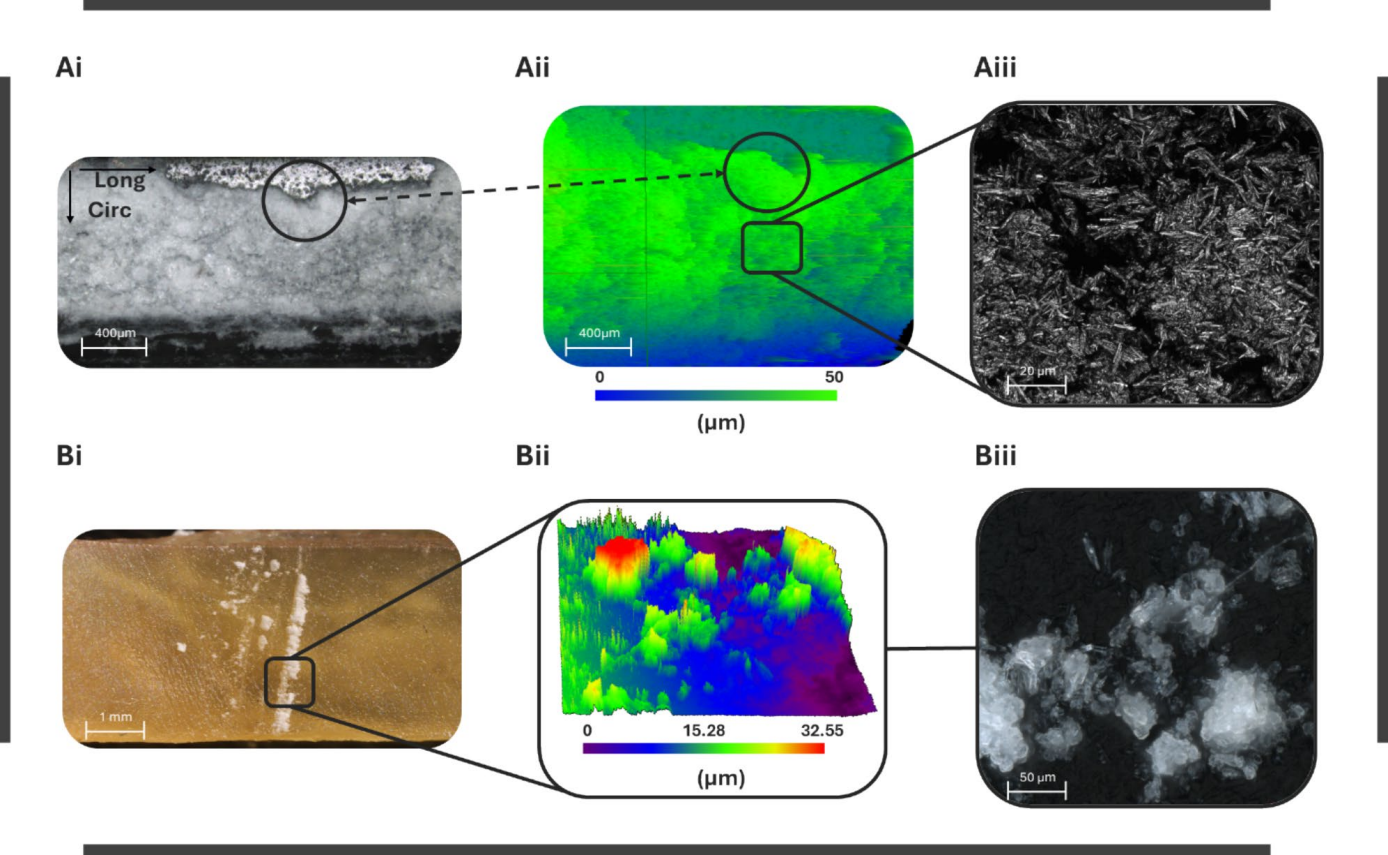
Coating characteristics pre- and post-stamping: **Ai** and **Aii** Coating thickness traced by the confocal laser microscope on the folded DCB device, **Aiii** coating microstructure under high-magnification (×100) confocal measurements, **Bi** coating transferred onto the arterial endothelium, imaged 3 days post-drying, **Bii** quantitative surface measurement of transferred coating reveals variability in coating transferred thickness, and **Biii** high-magnification (×50) confocal image of the transferred coating microstructure onto the artery

Confocal laser microscopy of the folded DCB indicated local coating thickness ranging between 32 and 48 μm (Fig. 8Ai and ii). High-magnification imaging revealed the microcrystalline structure of the coating (Fig. 8Aiii). Coating transferred to tissue demonstrated linearly patterned areas reminiscent of the coating onto the balloon prior to stamping (Fig. 8Bi). High-magnification (×50) confocal images of the local coating transferred onto arterial tissue revealed high variability in coating thickness, with a local maximum of 32.55 μm (Fig. 8Bii) and the transferred coating’s microstructure (Fig. 8Biii).

## Discussion

The known limitation of DCBs and their lack of a class effect creates the need for developing improved methodologies for preclinical models that realistically and controllably simulate the DCB–tissue interaction and allow studying and optimizing their coating delivery performance [9, 28]. Once coating delivery is predicted and optimized, it can be provided as input for drug elution studies to shed light on the therapeutic window that can exert suitable biological response. In vivo and ex vivo DCB inflations within vessels realistically simulate DCB–tissue interactions during treatment, providing a means to assess DCB performance. However, they are not suitable for controlled studies of coating transfer mechanisms. Additionally, each test condition (e.g., inflation pressure) requires a full device, necessitating a large number of devices for comprehensive DCB performance evaluation. Similarly, a large number of animals for in vivo tests or animal arteries for ex vivo inflation experiments are required, raising ethical concerns for in vivo studies and feasibility issues in obtaining appropriately sized arteries for ex vivo experiments. Conversely, benchtop coupon experiments are effective for investigating the impact of coating formulations and experimental variables on acute drug-coating delivery. Nevertheless, these present the major limitations of (i) the inability to evaluate commercial DCBs, hindering the head-to-head comparison, (ii) neglecting the mechanical straining and changes in coating microstructure of the drug coating during the folding, sheathing, unsheathing, and unfolding processes, (iii) neglecting possible changes in hydrophilicity during shelf life, (iv) disregarding the particulate generation prior to balloon–vessel interaction, and (v) lacking the proper replication of contact forces that occur at the balloon-tissue interface during in vivo DCB deployment. This necessitates developing a new experimental method that allows controlled testing of experimental variables while accurately replicating the DCB treatment.

This study aimed to create a parsimonious experimental method that simulates DCB–vessel interaction prior to DCB retraction and resumption of blood flow. This involved devising and implementing a pipeline that integrates multiscale in silico modeling and interfacial-level benchtop experiments using a commercial DCB as exemplification. Specifically, only two DCBs were exploited to perform the three pillars involved in the suggested analysis, where each one provides the required information to perform the subsequent. These involved PILLAR I. resin inflation and slicing, PILLAR II. device-level numerical simulations to deduce in vivo CPs and interfacial-level simulations to correlate these pressures with required ex vivo forces, and PILLAR III. ex vivo stamping experiments and image analysis. HPLC and confocal measurements were exploited in this study as additional tools to provide insights into the results.

Application of experimental activities involved in PILLAR I on the specific DCB proved the feasibility of resin inflation and subsequent slicing in DCB segments. Imaging of the resin-inflated DCB revealed highly heterogeneous coating on the balloon with stripes of dense coating, attributed to coating straining during manufacturing and DCB expansion (Fig. 3). Cylindrical DCB segments with repeatable dimensions and minimal coating loss during preparation were acquired (Fig. 3C and D). Since lateral portions of the DCB might have interacted with supports in PILLAR I and PILLAR III, stamping was performed at the central 5 mm of the stripe. Therefore, variability in coating distribution on the segments underscores the importance of imaging the coating onto the DCB prior to tissue interaction (Fig. 5). However, sample preconditioning was performed at the full length of the stripe to ensure complete coating engagement in the process.

Correlation of the DCB expansion and stamping simulations performed in PILLAR II with the specific DCB characteristics measured in PILLAR I demonstrated that the range of circumferential DCB-tissue contact W_c_ allowed for single stripe interaction for the selected testing forces F_st_, permitting exploitation of the 5 DCB stripes for 5 different experiments (Fig. 4).

Figure 5 demonstrates two characteristic cases of tests included in Group 2, where experimental analysis in PILLAR III revealed that coating is partially transferred in thickness at low CPs and is completely transferred in high CPs, being consistent with coating transfer by the cohesive or adhesive-cohesive coating failure limits outlined by Shazly et al. [29]. The data presented herein suggest that higher CPs can invoke balloon-coating adhesive failure and therefore complete coating transfer.

A strong correlation between Image analysis and HPLC data verifies the proposed methodology for quantitative evaluation of coating transfer and highlights the repeatability of the tests despite the heterogeneity of drug-coating onto the DCB (Fig. 6).

Both image and HPLC analysis revealed increasing coating/drug transfer with increasing average CP, which is consistent with literature [10, 11]. Increased DCB-to-lumen ratio can significantly increase interfacial CP in naïve vessels [14], and consequent drug transfer, as demonstrated in an ex vivo study [30]. Figure 7Ai demonstrates an initial linear increase in coating transfer area which then saturates with further increase in CP. HPLC data confirmed the linear increase in drug content from Low to Intermediate CP, while showcasing another 1.4-fold increase in drug transfer when increasing from Intermediate to High (Fig. 7Aii). The authors advocate that this increase was not captured by the image analysis due to its 2D limitation and hypothesize that thicker coating was transferred and was indented deeper at higher CPs as demonstrated by Lee et al. [13]. Considering maximum values of coating and drug quantity of each stripe at 14 mm^2^ (1.38 mm × 10 mm) and 118 μg [3538 μg total drug/(6 segments × 5 folds)], and assuming an equal distribution of drug and coating on the balloon, the balloons delivered 7–22% of coating and 4–152% of the drug to the tissue.

Stamping of preconditioned DCB segments (Group 2) demonstrated overall inferior transfer to tissue compared to pristine segment stamping (Fig. 7A and B) and an inverse trend with respect to pristine stamping. Notably, Low CP preconditioned DCB segments transferred higher coating area and drug quantity during subsequent stamping at High CP when compared to segments preconditioned with High CP. This verifies the role of higher CP in transferring increased drug coating. Additionally, this underscores the necessity of having information about the coating condition on the DCB prior to stamping and enhances the potential of the adopted approach, as this cannot be obtained during DCB inflation inside vessels. Assuming an equal distribution of drug and coating in the whole DCB, the balloons delivered 2–16% coating and 13–135% drug onto the tissue, respectively. The drug quantity transferred during pristine stamping (Group 1) was roughly half of the one transferred during preconditioning (Group 2) attributed to the 1:2 contact area ratio and signifying that contact time has an insignificant role in drug-coating transfer for the specific device (Fig. 7A and B). Correspondingly, slightly reduced coating area transfer in the case of preconditioning is attributed to the involvement of the full (partially damaged) stripe engagement during preconditioning.

Preconditioning via 2 s stamping (Fig. 7C) shows a linear increase in coating transfer area from Low to Intermediate CP, which is confirmed by HPLC data and subsequent saturation when increasing from Intermediate to High, while HPLC continues to show a linear increase in drug content (Fig. 7Bi and ii), just like in the case of pristine stamping. This strengthens the inference that the image analysis of the tissue surface is unable to capture the increase observed by HPLC measurement, which also quantifies subsurface drug in the tissue. Notably, the comparable outcomes between short-term compression and prolonged stamping indicate that brief durations are as effective in transferring the drug coating to tissue as extended periods. This suggests that the specific DCB could achieve equivalent drug delivery in naïve vessels when inflated for either 2 or 60 s. Assuming an equal distribution of drug and coating, the balloons delivered 1–34% coating and 4–305% drug onto the tissue.

Transfer of coating onto arterial tissue showed significant thickness variability, likely stemming from non-uniformity in coating thickness on the balloon prior to tissue interaction (caused by straining), local coating cracking during compression, or local differences in coating micromechanical pressures (Fig. 8Bii). Future studies can benefit from more rigorous confocal measurements to establish a correlation between CP exerted onto the tissue and coating volume fraction transferred onto the tissue.

Our study focused on the acute transfer of the coating during DCB deployment, which is a critical precursor to drug transport, and subsequent analysis of bulk drug delivered. This aspect has been investigated by many studies. For example, Seidlitz et al. [9] developed an in vitro setup to estimate drug loss during DCB advancement and transfer to a simulated vessel wall. Their study found that only about 1% of certain drugs were transferred, largely due to mechanical instability and coating loss during transit. Kempin et al. [31] also explored this by testing different paclitaxel (PTX) coatings, finding that solvent choice and the use of additives like polyvinylpyrrolidone significantly affected drug transfer rates, with commercial DCBs like SeQuent Please achieving a transfer rate of about 17%. Petersen et al. [8] evaluated various coating methods and compositions, finding that pipetting processes with specific PTX formulations resulted in more consistent drug transfer, with up to 40% of the drug being transferred during balloon inflation. Additionally, in an in vivo animal study by Tzafriri et al. [10] reported that 10–17% of the coating was transferred to the vessel wall during DCB deployment. Our study observed coating transfer rates between 7 and 22% for pristine DCB segments and 2–16% for preconditioned segments, with corresponding drug transfer rates ranging from 4–152% to 13–135%. These results align with the ranges reported in the literature, indicating that while there is variability based on the DCB formulation and preparation, our approach is consistent with current findings.

The therapeutic benefits of DCB coatings are determined by the amount of drug that is successfully transferred to the arterial wall and retained over time. The level of drug required to exert a biological effect, such as inhibiting neointimal hyperplasia or restenosis, is influenced by several factors, including the drug type, concentration, coating technology, and the specific vascular bed treated. Studies underscore that while the precise threshold may vary depending on the specific clinical scenario and drug formulation, the goal is to achieve sufficient local drug concentration that remains above the therapeutic threshold for a duration adequate to exert the desired antiproliferative effect. In vitro experiments with PTX demonstrated that smooth muscle cell (SMC) proliferation could be effectively suppressed when the IC50 of PTX was 1–2 ng/g, while SMC migration could be inhibited at an IC50 of 0.4 ng/g [32–34]. Extrapolation of these in vitro estimates to the in vivo setting remains a matter of debate owing to the biological differences between in vitro and in vivo cells and the inertness of solid paclitaxel prior to its solubilization as well as their inhomogeneous distribution within the tissue. Computational modeling of contact pressure [14] correlated with local coating transfer lays the foundation for computationally predicting coating pattern distribution onto the tissue. By integrating this acute distribution model with equations that account for local coating dissolution, diffusion of solubilized drug, and binding to receptors [35, 36] predictions of local effects can be achieved [37]. To this end, the current study aimed to quantify the dependence of coating transfer on contact pressures under controlled conditions.

This study presented a number of limitations and assumptions. Firstly, coating detachment during DCB sample preparation was not quantified, preventing us from evaluating the impact of preparation on coating loss. However, in clinical practice, balloons will experience similar straining during unsheathing, handling, and tracking prior to DCB expansion. Additionally, to overcome the challenge posed by coating variability along the DCB segments, we focused our analyses on areas within a single densely coated stripe. Though distribution of coating in these areas of focus was not perfectly homogeneous, 4 randomized tests were performed for each condition. Nevertheless, variability in drug-coating transfer to tissue observed with four tests per experimental variable suggests that increasing the number of full DCB devices could enhance the statistical significance of the results while maintaining methodological efficiency. The infusion method we employed also had limitations and did not allow for application of infusion pressures above 1.8 atm due to resin leakage. To replicate in vivo conditions more accurately, further enhancements are necessary to permit higher inflation pressures and to incorporate the effects of balloon stretching on DCB segments, such as potential coating cracks, as well as the inclusion of blood flow. The use of aortic tissue instead of peripheral arteries was driven by practical considerations and the lack of available peripheral samples. The aortic tissue provides advantages such as minimizing curvature effects and reducing substrate interference and has been adopted in stamping experiments [16]. We do not expect this consideration to significantly impact our findings, as our focus was on the interfacial interaction between the DCB coating and the endolumen, with structural differences accounted for in our numerical simulations. Artery material in the stamping simulation was modeled as elastic and isotropic, which simplifies the complex, multilayered structure of the arterial wall. While this approach is supported by several studies [24, 25, 38, 39], it does not capture the distinct mechanical properties of the individual arterial layers. Although our sensitivity analysis showed that varying vessel stiffness had no significant impact on the results, we acknowledge that a more detailed, multilayered model would be necessary for studies aimed at determining exact CP values and more accurately simulating the arterial wall's mechanical behavior.

In conclusion, the presented and implemented computational-benchtop approach to test the influence of balloon–vessel interaction on coating transfer from commercial DCBs enhances the realism of published benchtop models. The novelties introduced included (i) use of commercial devices for stamping experiments instead of in-house coupons allowing head-to-head device comparison, (ii) efficient device exploitation, maximizing the number of tests and investigated conditions per device while minimizing the cost, (iii) replicating controlled in vivo conditions during DCB–artery interaction, (iv) elucidation of coating’s condition post-inflation and pre-compression onto tissue, and (v) employing a novel and simple image analysis for coating transfer assessment. HPLC measurements confirmed a correlation between higher CP and increased drug content. For the tested DCBs, results indicate that increased acute coating transfer correlates with higher CP, consistent with the literature. Lower CPs showed partial coating transfer, suggesting a correlation between CP and coating transfer thickness. Reduced transfer after initial coating preconditioning underscores the importance of coating condition prior to stamping, while DCB stripes preconditioned with higher CP delivered lower coating in further High CP stamping. Overall, our findings demonstrate the feasibility of this approach and highlight its potential for accelerating the development of future DCB technologies, comparing relative performances of competing technologies, and optimizing treatment variables for improved drug delivery. These results can now be used to predict coating distribution in treated arteries, providing a foundation for developing a computational 3D model of drug distribution and receptor binding at arterial treatment sites.

## Supplementary Information

Below is the link to the electronic supplementary material.

Supplementary file1 (PDF 200 kb)
