# Supplementary material for: A Novel In Silico–Ex Vivo Model for Correlating Coating Transfer to Tissue with Local Drug-Coated Balloon-Vessel Contact Pressures
Source: Ann Biomed Eng. 2024 Dec 12;53(3):740–57. doi: 10.1007/s10439-024-03634-6 (PMC11836097; doi:10.1007/s10439-024-03634-6)
Supplement: Supplementary file 1 — Supplementary file1 (PDF 200 kb) [file 10439_2024_3634_MOESM1_ESM.pdf]

Supplementary material

Impact of Vessel Pressurization on Contact Pressure Distribution

To explore the influence of vessel pressurization on DCB deployment, we conducted additional simulations with the vessel pressurized to 100 mmHg prior to balloon expansion. These results were then compared to those obtained from the original simulations, where the vessel was unpressurized. Our analysis showed that the overall patterns of contact pressure distribution were consistent between the pressurized and unpressurized scenarios, indicating that the primary interaction dynamics were preserved. However, there was a slight decrease in the average contact pressure values in the pressurized vessel, due to the vessel's reduced radial resistance when pressurized, which allowed it to deform more easily during DCB deployment. Despite these changes in average pressure values, the range of contact pressures remained roughly the same. These findings suggest that while vessel pressurization does influence the specific values of contact pressure, it does not significantly alter the overall pressure range experienced during DCB deployment.

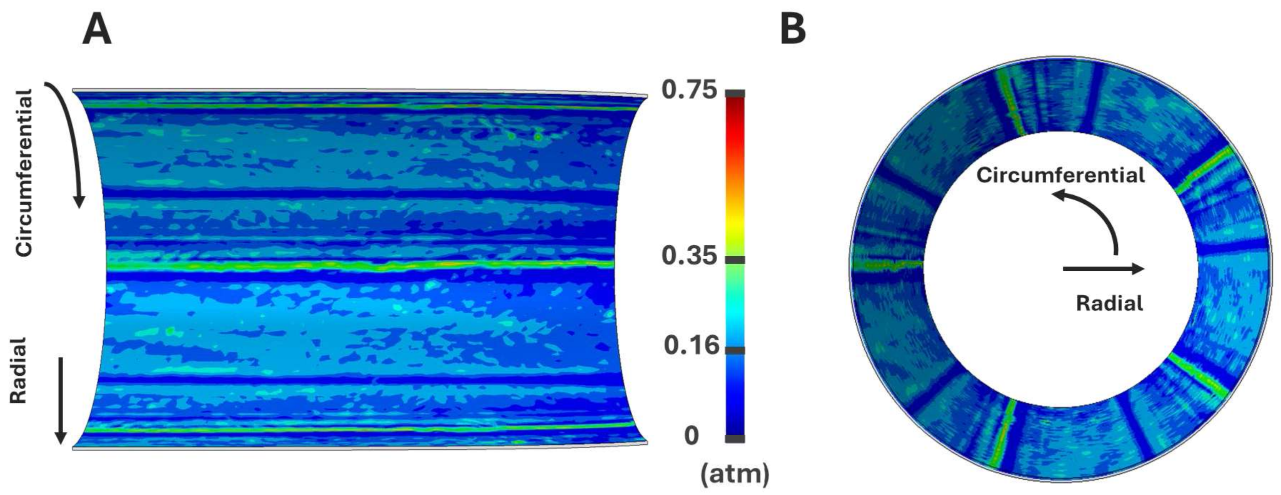

Supplemental Fig 1. Numerical simulations of DCB expansion inside a pressurized vessel at 100mmHg at 9 atm of balloon inflation pressure A. Longitudinally sliced section of the arterial endolumen showing predicted CP and B. Perspective side view of the treated area.
